# Modeling and Predicting Seasonal Influenza Transmission in Warm Regions Using Climatological Parameters

**DOI:** 10.1371/journal.pone.0009450

**Published:** 2010-03-01

**Authors:** Radina P. Soebiyanto, Farida Adimi, Richard K. Kiang

**Affiliations:** 1 Global Change Data Center, NASA Goddard Space Flight Center, Greenbelt, Maryland, United States of America; 2 Goddard Earth Science and Technology Center, University of Baltimore County, Baltimore, Maryland, United States of America; 3 Wyle International, McLean, Virginia, United States of America; University of Liverpool, United Kingdom

## Abstract

**Background:**

Influenza transmission is often associated with climatic factors. As the epidemic pattern varies geographically, the roles of climatic factors may not be unique. Previous *in vivo* studies revealed the direct effect of winter-like humidity on air-borne influenza transmission that dominates in regions with temperate climate, while influenza in the tropics is more effectively transmitted through direct contact.

**Methodology/Principal Findings:**

Using time series model, we analyzed the role of climatic factors on the epidemiology of influenza transmission in two regions characterized by warm climate: Hong Kong (China) and Maricopa County (Arizona, USA). These two regions have comparable temperature but distinctly different rainfall. Specifically we employed Autoregressive Integrated Moving Average (ARIMA) model along with climatic parameters as measured from ground stations and NASA satellites. Our studies showed that including the climatic variables as input series result in models with better performance than the univariate model where the influenza cases depend only on its past values and error signal. The best model for Hong Kong influenza was obtained when Land Surface Temperature (LST), rainfall and relative humidity were included as input series. Meanwhile for Maricopa County we found that including either maximum atmospheric pressure or mean air temperature gave the most improvement in the model performances.

**Conclusions/Significance:**

Our results showed that including the environmental variables generally increases the prediction capability. Therefore, for countries without advanced influenza surveillance systems, environmental variables can be used for estimating influenza transmission at present and in the near future.

## Introduction

Influenza remains a global concern as estimates show that the annual epidemics may cause up to five million severe illnesses and 500,000 deaths worldwide [1]. Vaccination strategy and surveillance effort as means of prevention prevail only in temperate regions, especially in the northern hemisphere. Many countries in the subtropics and tropics, on the other hand, underutilize the prevention mechanisms [2] despite the year-round outbreaks and recent findings that (i) East and Southeast Asia act as a source of new influenza strains and transmission [3], [4], and (ii) epidemics timing in South America travels southward starting from the equator [5], [6]. The scarce surveillance data and the spatiotemporally varying transmission pattern further complicate the development of appropriate vaccination for tropical regions. The tropics consist of the equatorial regions between the Tropic of Cancer (23.4°N) and the Tropic of Capricorn (23.4°S). The regions next to the tropics with latitude less than 40°N and greater than 40°S are the subtropics. Because in this analysis the same reasoning is applicable to both the tropics and the subtropics, for brevity henceforth the term tropics will include subtropics as well.

Unlike influenza transmission in the tropics that greatly varies geographically, the consistent wintertime influenza peak in temperate regions is often associated with, arguably, the corresponding dry and cold climate [7], [8]. It is not only a condition in which aerosol-borne influenza transmission is most favorable [9], [10], but also one that promotes indoor crowding tendency which may lead to higher risk for contact transmission [11]. In addition to temperature and humidity, the El Nino Southern Oscillation (ENSO) [12] and solar radiation [13] has been implicated in influenza transmission in temperate climate. On the other hand, the role of climate on influenza in the tropics is much less understood. Several regions observe high influenza transmission that coincides with rainy season such as southern India, Vietnam and Brazil [14], [15], [16], [17]. While others such as Singapore, Thailand and Philippines, detect semi annual peaks that are not necessarily associated with rainfall [14]. *In vivo* study by Lowen et al. [18] emphasized the dominating effect of contact transmission in the tropics, while Alonso et al. [5], on the contrary, showed that temperature and humidity contribute more to the southward influenza spread in Brazil than contact transmission. Thus it appears that the contributing factors to influenza transmission in the tropics are region-specific due to the highly varying transmission pattern.

With the scarcely-available surveillance data for the tropics, we chose to study influenza transmission pattern in two regions that, similar to the tropics, are characterized by warm climate, but have advanced influenza surveillance system. These regions are Hong Kong, China (22° N) and Maricopa County, Arizona (33° N)–with Hong Kong in the tropics and Maricopa County in the subtropics. The objective of the study was to investigate and model the effect of climate on the transmission pattern. The resulting model, in turn, can be employed to forecast influenza epidemics in the tropics that may help to facilitate vaccination strategy development and antiviral distribution. Furthermore, the use of climatic parameters in the forecast for tropics is highly advantageous not only due to few studies focusing on the influenza prediction–as demonstrated by Viboud et al. [19])–but also due to the low surveillance efforts in these regions.

## Results

Throughout this study, we utilized a time series-based model namely Autoregressive Integrated Moving Average (ARIMA), as well as SARIMA when seasonality is included. The method is briefly delineated under Methods section. In the following, results for influenza Hong Kong will be presented first, followed by Maricopa County results.

### Hong Kong

**Figure 1:**
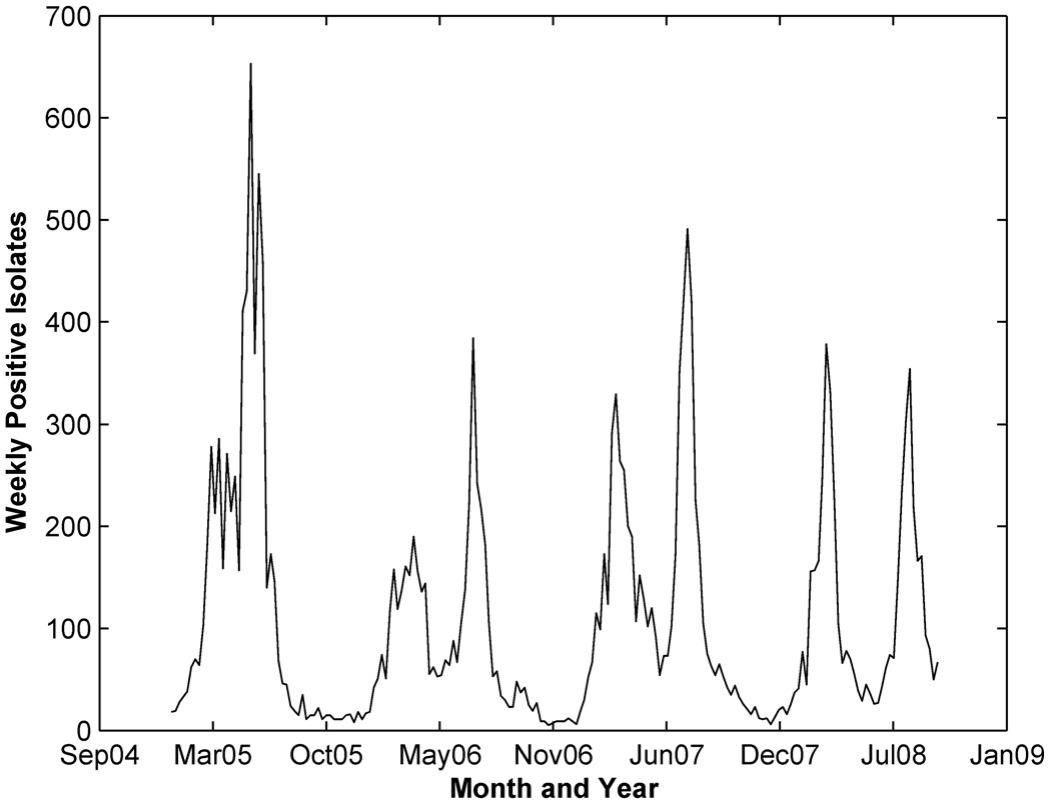
Hong Kong weekly influenza positive isolates.

As a first step in ARIMA modeling, we seek to stationarize the response series, which is the influenza weekly count in Hong Kong shown in Figure 1. Taking the log transformation of the series reduced the variances of the influenza cases, and subsequent differencing–either first or seasonal order of difference–resulted in stationary series. We further used the Auto-Correlation and Partial Auto-Correlation Function (ACF and PACF, respectively) plots to identify the order of the ARIMA model for the stationary series. For the first-order differenced series, both the ACF and PACF cut off at lag 2, whereas for the seasonally differenced series the ACF decreases very slowly with PACF cut off at lag 2.

We further fit several univariate (S)ARIMA models with different orders, and consequently excluded any models in which the residuals exhibit autocorrelation. The resulting models and the estimated coefficients are summarized in the top half of Table 1 (Please see Methods section for ARIMA model notation). As we can see in Table 1, ARIMA(2,1,2) has the best Root Mean Square Error (RMSE) for the fitted dataset, ARIMA(1,1,2) has the best predictive RMSE, while SARIMA(2,0,0)(0,1,0) has the lowest Akaike Information Criterion (AIC). Among these four univariate models, the relative differences of the worst from the best performing AIC is 6%, fit RMSE is 27% and prediction RMSE is 16%. Since the AIC differences is relatively low, and the prediction RMSE not only have smaller differences but also it is unavailable in a realistic case, we will henceforth use the model with smallest fit RMSE–that is ARIMA(2,1,2)–as a baseline univariate model for further comparison.

**Table 1:** Summary of model performance and the estimated coefficients for Hong Kong Influenza.

Model	Fit		Prediction	AR		MA		Environmental variables		
	RMSE	AIC	RMSE	Est.	Pr > |t|	Est.	Pr >|t|	Vars	Est.	Pr >|t|
ARIMA(2,1,2)	0.4045	166.26	0.4788	0.44	<.0001	0.588	0.0037			
				−0.446	<.0001	−0.785	0.0025			
ARIMA(1,1,2)	0.4071	166.25	0.4321	0.45	0.0226	0.603	0.0014			
						−0.375	<.0001			
SARIMA(1,0,0)(0,1,0)	0.5144	159.46	0.4993	0.774	<.0001					
SARIMA(2,0,0)(0,1,0)	0.5074	156.56	0.5033	0.608	<.0001					
				0.219	0.0288					
ARIMAX(1,1,2) with LST, RF, and RH	0.3675	138.51	0.5292	0.426	0.0181	0.6443	0.0001	LST (Lag2)	−0.035	0.0016
						−0.446	<.0001	LST (Lag5)	−0.0307	0.0049
								RF (Lag 3)	0.0534	0.0047
								RH	0.0164	<.0001
SARIMAX(0,1,2)(1,0,0) with LST, RF and RH	0.3662	137.52	0.5433	0.276	0.0104	0.2937	0.0005	LST (Lag2)	−0.036	0.0011
						−0.316	0.0002	LST (Lag5)	−0.0324	0.0032
								RF (Lag 3)	0.0527	0.0064
								RH	0.0168	<.0001
SARIMAX((2),1,0) with LST	0.4013	156.76	0.4649	0.251	0.0022			LST (Lag2)	−0.028	0.014
								LST (Lag5)	−0.0256	0.0257
SARIMAX(1,0,0)(0,1,0) with LST	0.4666	134.7	0.5104	0.795	<.0001			LST (Lag2)	−0.048	0.0009
								LST (Lag5)	−0.0312	0.0248
ARIMAX(2,1,0) with RF	0.4174	168.62	0.4029	0.244	0.0017			RF (Lag 3)	0.0244	0.1985
ARIMAX((2),1,0) with RH	0.4073	163.34	0.4728	0.247	0.0021			RH (Lag 1)	0.011	0.0055
SARIMAX(1,0,1)(0,1,0) with RH	0.4968	152.76	0.5831	0.883	<.0001	0.2588		RH (Lag1)	0.0144	0.0086
ARIMAX((2),1,0) with LST and RH	0.3872	148.07	0.5273	0.259	0.0018			LST (Lag2)	−0.029	0.0096
								LST (Lag 5)	−0.029	0.0097
								RH (Lag 1)	0.0124	0.0013

Abbreviations: ARIMA  =  Autoregressive Integrated Moving Average; S = Seasonal; X = with external/input series; LST  =  Land Surface Temperature; RF  =  Accumulated Rainfall; RH  =  Relative Humidity; RMSE  =  Root Mean Square Error; AIC  =  Akaike's Information Criterion; AR  =  Autoregressive coefficients; MA  =  Moving Average Coefficients; Est  =  Estimated values through conditional least square method.

In order to include the environmental parameter as input series to the model, we first examined the correlations between the influenza cases and the environmental series. Our results (Table 2) show that there are significant correlations (based on the two standard error limits) with LST at lag 2 and 5, rainfall at lag 3 and relative humidity at lag 0 to 3. Subsequently (S)ARIMA models were estimated with one or more environmental variables included. The performances of these models and the estimated coefficients are shown in Table 1. For these multivariate models, the best fit RMSE is obtained from SARIMAX(0,1,2)(1,0,0) with LST, rainfall and relative humidity as covariates. SARIMAX(1,0,0)(0,1,0) with LST has the best AIC, while ARIMAX(2,1,0) with relative humidity has the best prediction RMSE. Comparing these three models with the baseline univariate model discussed previously (ARIMA(2,1,2)), we found that including the environmental input series improve the AIC by 18%, the fit RMSE by 9% and the prediction RMSE by 16% from the baseline model.

**Table 2:** Hong Kong: Cross-correlations between pre-whitened environmental series and the influenza counts.

Variable	Lag							
	0	1	2	3	4	5	6	7
LST	0.0515	0.0083	−0.1835*	−0.1247	−0.1132	−0.2643*	−0.0638	−0.1572
RF	0.0005	−0.0063	0.1211	0.1417*	0.1045	0.1071	0.0960	0.0532
RH	0.1358*	0.2717*	0.1496*	0.1463*	0.0578	0.0745	0.0730	0.0359

*indicates significant at the two-standard error. See **Table 1** caption for abbreviations.

**Figure 2:**
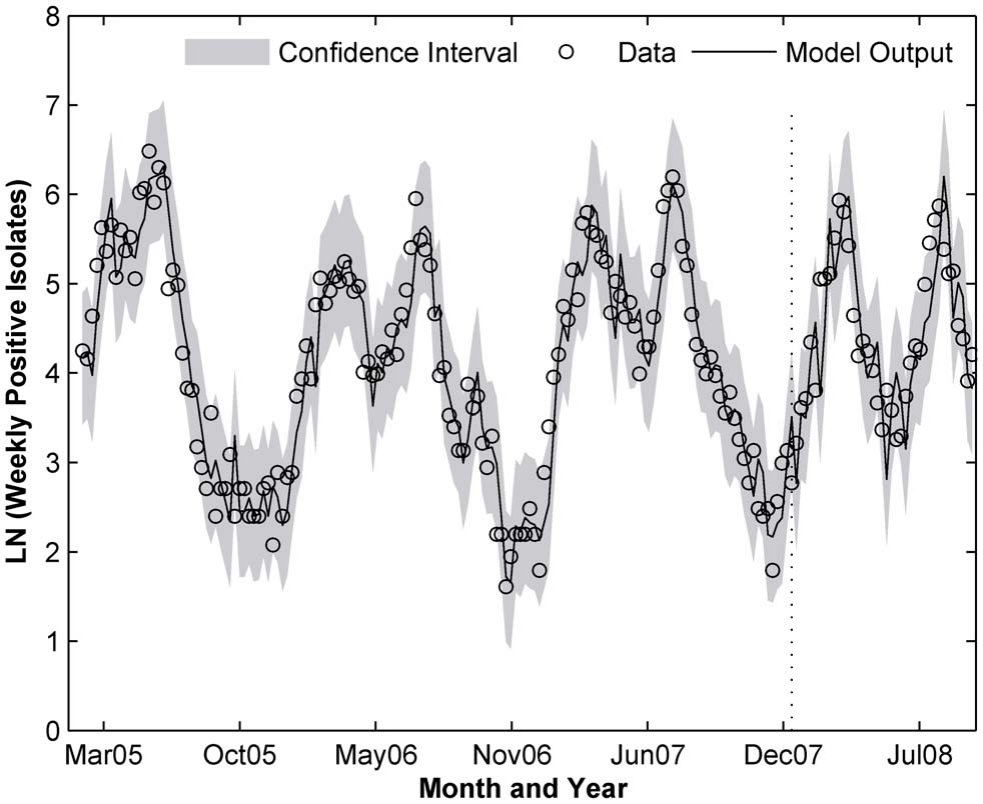
Hong Kong fitted and predicted values as separated by the dashed line. SARIMAX(0,1,2)(1,0,0) with LST lag 2 and 5, accumulated rainfall lag 3 and relative humidity lag 1.

**Figure 3:**
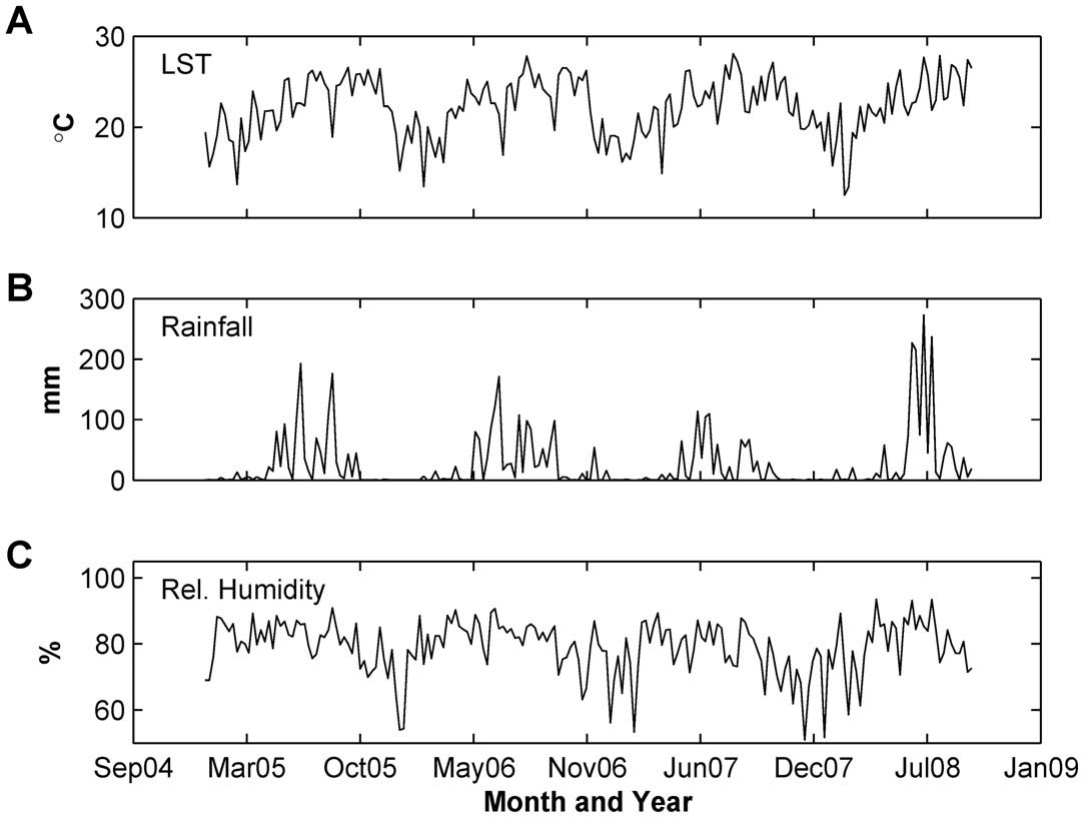
Hong Kong environmental variables. (A) weekly average of Land Surface Temperature (LST), (B) weekly accumulated rainfall and (C) weekly average of mean relative humidity.

Among the three best performing multivariate models, ARIMAX(2,1,0) with rainfall as input, has the highest AIC values. Moreover, it has high p-value for estimated rainfall coefficient in this model (Table 1). Thus we can exclude this model from the selection. Between SARIMAX(1,0,0)(0,1,0) with LST only and SARIMAX(0,1,2)(1,0,0) with LST, rainfall and relative humidity, the differences in AIC is small (2%), but there is about 27% differences in the fit RMSE. Thus we choose the SARIMAX(0,1,2)(1,0,0) with LST, rainfall and relative humidity since it has the lower fit RMSE. The fitted and predicted values of this model were plotted in Figure 2, and the associated environmental variables are shown in Figure 3.

### Maricopa County

**Figure 4:**
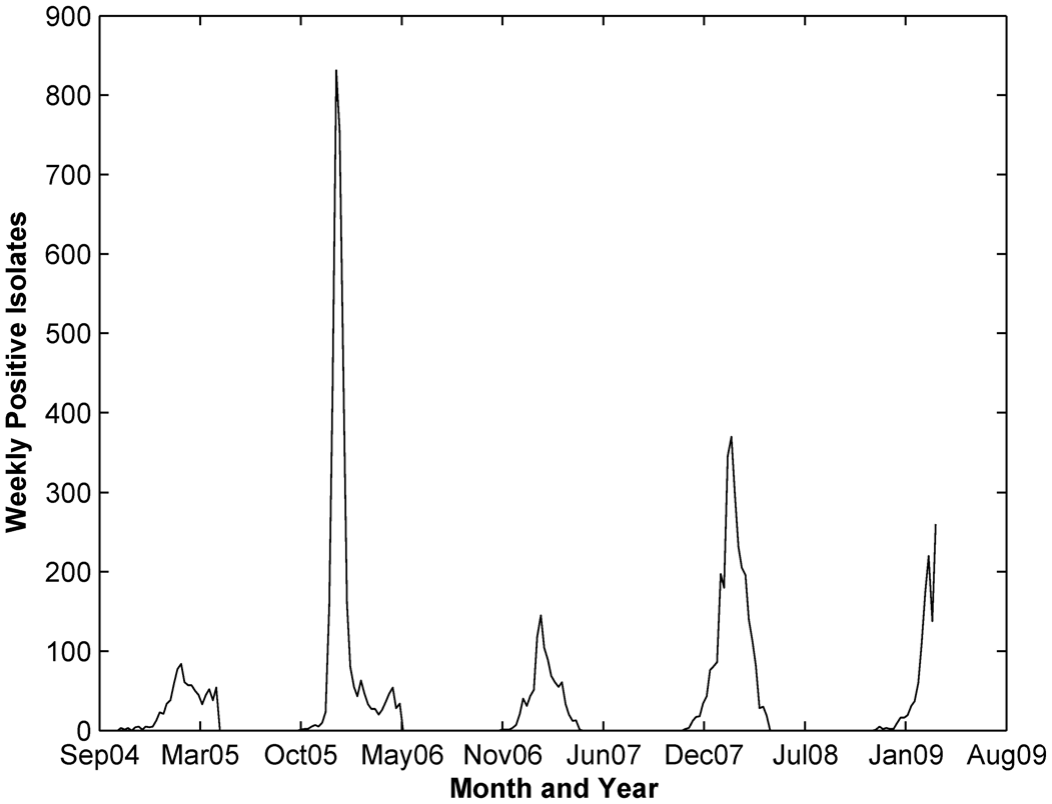
Maricopa county influenza cases.

Figure 4 showed the time series profile for positive influenza counts in Maricopa County. Similar to Hong Kong influenza, taking the log transformation of the series reduced the variation in the variances. Stationarity of the log-transformed series was achieved through first order differencing. Further plotting the ACF and PACF of the stationary series reveals significant autocorrelations at lag 2 and 21, and significant partial autocorrelation at lag 2. Based on this ACF and PACF, we fitted several univariate (S)ARIMA models and found that the two best performing models are SARIMA(1,1,0)(1,0,0) and SARIMA(0,1,0)(1,0,0). As shown in Table 3, the performances of the univariate models measured by AIC and RMSE for the fitting dataset were similar. Thus we chose SARIMA(0,1,0)(1,0,0) as the baseline model because all p-values of the estimated coefficient are relatively significant (<0.05).

**Table 3:** Summary of model performances and the estimated coefficients for Maricopa County influenza.

Model	Fit		Pred.	AR		MA		Enviromental vars		
	RMSE	AIC	RMSE	Est.	Pr >|t|	Est.	Pr >|t|	Vars	Est.	Pr >|t|
SARIMA(0,1,0)(1,0,0)	0.5862	280.582	0.5123	0.34964	<.0001					
SARIMA(1,1,0)(1,0,0)	0.5859	282.454	0.5107	0.02694	0.7228					
				0.34854	<.0001					
SARIMAX(1,0,1)(0,1,0) with LST	0.6087	196.920	0.6063	0.86496	<.0001	−0.20367	0.062	LST (Lag 3)	−0.0418	0.004
SARIMAX(2,0,0)(0,1,0) with T_mean_	0.5493	170.958	0.6058	1.2497	<.0001			Tmean (Lag 1)	0.0188	0.012
				−0.37896	0.0001			Tmean (Lag 7)	0.015	0.044
SARIMAX(1,0,0)(0,1,0) with RH_Max_	0.6238	207.660	0.6142	0.9321	<.0001			RhMax (Lag 3)	0.0101	0.022
								RhMax (Lag 6)	0.0071	0.093
SARIMAX(1,0,0)(0,1,0) with RH_Min_	0.6382	210.540	0.5867	0.8979	<.0001			RHMin (Lag 6)	0.0173	0.047
SARIMAX(0,1,0)(1,0,0) with P_max_	0.5370	247.390	0.5101	0.41313	<.0001			Pmax (Lag 0)	−0.016	0.043
								Pmax (Lag 6)	−0.0126	0.106
ARIMAX(1,0,0) with LST and RH_Max_	0.5753	277.840	0.5522	0.90303	<.0001			LST (Lag 3)	−0.073	<.0001
								RHMax (Lag 3)	−0.013	0.004
SARIMAX(1,0,0)(0,1,0) with LST and RH_Min_	0.6048	195.727	0.6137	0.91289	<.0001			LST (Lag 3)	−0.0413	0.004
								RHMin (Lag 6)	0.0197	0.023

Abbreviations: T_mean_  =  mean air temperature; RH_Max_  =  Maximum Relative Humidity; RH_Min_  =  Minimum Relative Humidity. See Table 1 for other abbreviations.

**Figure 5:**
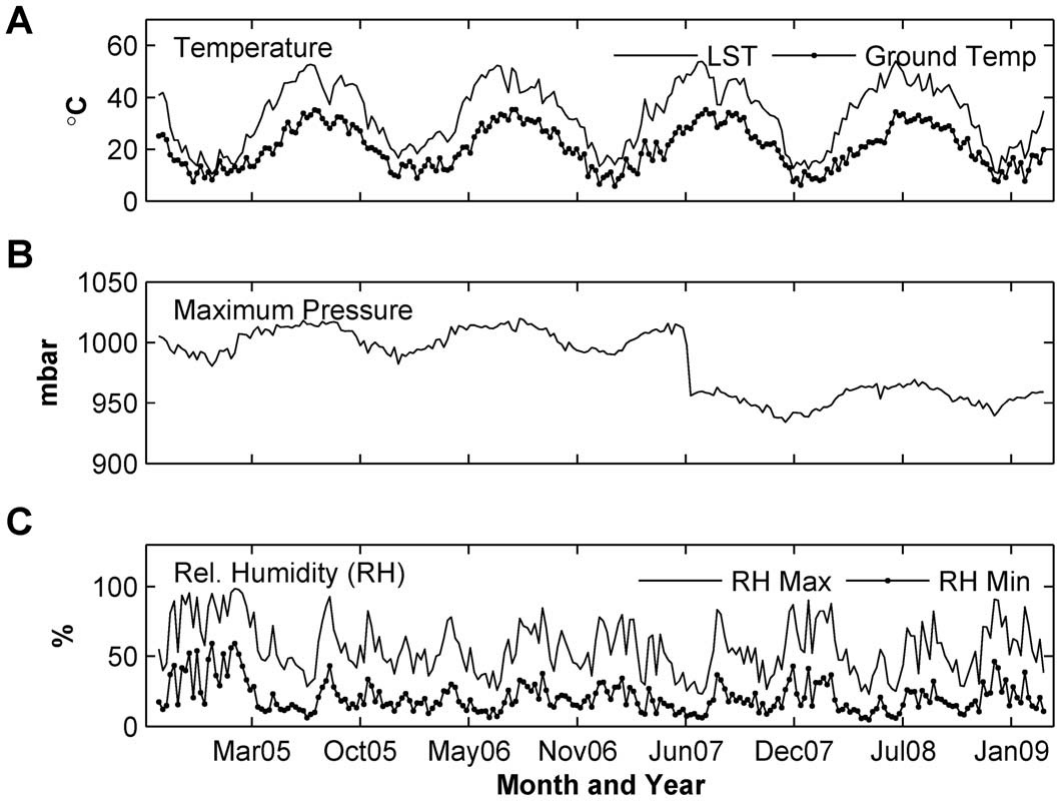
Maricopa environmental variables. (A) LST weekly average and Mean Temperature from ground station, (B) weekly average maximum pressure and (C) weekly average of minimum and maximum relative humidity.

We further pre-whitened the environmental variables and calculated the cross-correlation function. Variables that exhibit significant cross-correlations are shown in Table 4. As we can see, influenza cases in Maricopa County are correlated with LST at lag 3, mean air temperature at lag 1 and 7, maximum relative humidity at lag 3 and 6, minimum relative humidity at lag 6, and maximum air pressure at lag 0 and 6. The time series profiles of these variables are illustrated in Figure 5.

**Table 4:** Maricopa county: cross-correlations between influenza count and pre-whitened environmental series.

Variable	Lag							
	0	1	2	3	4	5	6	7
LST	0.0482	0.1173	0.0242	−0.1795*	0.0250	−0.0043	−0.0648	0.1706
T_mean_	0.1432	0.1836*	0.0405	0.0054	0.0468	0.0328	0.0188	0.1811*
RH_Max_	−0.0854	−0.1948	0.0010	0.2555*	−0.0839	0.1655	0.2461*	0.0806
RH_Min_	0.0055	−0.0634	−0.0411	0.1408	−0.0489	0.0378	0.2378*	0.1155
P_max_	−0.1368*	−0.0275	0.0008	−0.0540	−0.0809	−0.0251	−0.1418*	−0.0305

*indicates significant at the two-standard error. See **Table 3** caption for abbreviations.

**Figure 6:**
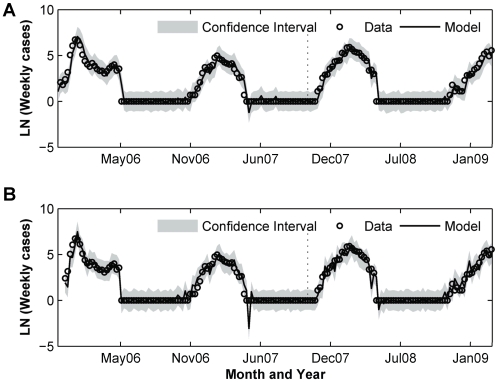
Maricopa County fitted and predicted values–as separated by the dashed line–from (A) SARIMAX with T_mean_ lag 1 and 7, (B) SARIMAX with P_max_ lag 0 and 6.

We further fitted (S)ARIMAX model with the lagged environmental variables as input series, and the results are summarized in Table 3. Overall, including one or more input series improves the model performances as compared to the baseline univariate SARIMA model previously described. Note that we kept some of the input variables even though the estimated coefficients have p-values greater than 0.05. This is mainly due to the model residuals that exhibit autocorrelation when these variables are removed. Our results indicate that incorporating the mean air temperature (T_mean_) at lag 1 and 7 yield the lowest AIC value for the fitting dataset. As compared with the baseline univariate SARIMA model previously described, the addition of T_mean_ improves the AIC by 39% and the RMSE by 6%. Lowest RMSE for both fitting and prediction dataset is obtained by SARIMAX model with maximum air pressure (P_Max_) at lag 0 and 6 as covariates. Compared with the baseline univariate SARIMA, the addition of P_Max_ improve the fit and predicted RMSE by about 9% and 0.4%, respectively. We showed in Figure 6 the fitted and predicted values produced by SARIMA model with T_mean_ and P_max_.

## Discussion

Through the use of ARIMA models, we explored the relationship between environmental variables and influenza cases in two regions characterized by warm climate, Hong Kong (China) and Maricopa County (Arizona, USA). We first examined whether influenza cases can be modeled as a univariate (S)ARIMA where it only depends on its own past values and random errors. We found that the univariate ARIMA was capable of forecasting 1-step ahead future influenza cases relatively well. For Hong Kong influenza, the best univariate model is ARIMA(2,1,2), where influenza cases depend on cases in previous two weeks. While for Maricopa County, the best univariate model is the seasonal SARIMA(0,1,0)(1,0,0), which depends on the cases in the previous one season. In the time series plot of influenza cases in Maricopa County (Figure 4), the seasonality is very distinct compared to influenza in Hong Kong (Figure 1). Thus it is expected that influenza cases in Maricopa County are modeled best when seasonality is incorporated.

In the multivariate ARIMA models, we found that the accumulated rainfall, land surface temperature (LST) and relative humidity are significant predictors for influenza in Hong Kong. The association of rainfall with influenza is commonly observed in tropical countries such as in Brazil [17], Singapore [15] and Thailand [16]. There is yet any direct relationship connecting rainfall with either the effectiveness of influenza transmission, virus survivorship or host susceptibility. Common observation is that rainfall may cause changes in the social behavior that in turn promotes contact transmission. For example, in rainy seasons, more people prefer indoor activities that may increase the chance for social contact, and hence contact transmission. In Hong Kong, rainy season is between April to September, with heavy and persistent rain typically in May and August. Meanwhile, influenza transmission typically peaks around March–April, and June–August (Figure 1), which in general is considered to be rainy season.

Temperature and relative humidity is often associated with influenza epidemics such as in Tokyo (Japan) [8] and especially in temperate regions where influenza peak coincides with winter. The prevailing dry and cold condition during winter seems to enhance influenza transmission, though this not substantiates high influenza transmission in the tropics. Lowens et al [9] conducted *in vivo* study and found that at low temperature (5°C) and low relative humidity (20% to 35%), transmission through aerosol is most efficient. At 20°C, aerosol transmission efficiency varies with relative humidity [9]. On the other hand, 30°C blocks aerosol transmission but not contact transmission, which explains influenza transmission in the tropics [18]. In our Hong Kong analysis, land surface temperature (LST) is used instead of air temperature, but LST can be used as a proxy for the other.

The first influenza peak in Hong Kong typically occurs During March–April, when the temperature starts rising. The normal mean temperature at this time is 18–22°C, whereas the relative humidity is 75%–80%. According to Lowens et al. [9], 20°C temperature and a relative humidity of 65% induce relatively higher transmission, whereas no transmission occurs at 80% relative humidity. Thus Hong Kong condition in March–April, lies between intermediate to no transmission zone as concluded by Lowens et al. [9]. However note that during this time of the year, rainfall starts to increase as well. As a result, the combination of moderate aerosol transmission efficiency as determined by temperature and relative humidity, and the behavioral response that promote contact transmission due to rainfall, causes high transmission rate during this time of the year. The second peak of the influenza transmission, on the other hand, occurs during summer where mean temperature is normally between 26–28°C and relative humidity is above 80%. At this condition, aerosol transmission is theoretically blocked, but the high rainfall frequency promotes the risk of contact transmission. Thus it seems that the second influenza peak is predominantly caused by the contact transmission.

Our results for influenza in Maricopa County showed that influenza cases are correlated with air and land surface temperature, maximum air pressure, and both maximum and minimum relative humidity (Table 3). As we have previously discussed, low temperature and relative humidity induce aerosol transmission. However, the best model is obtained when only maximum pressure is included as input series (Table 4). Air pressure is an important determinant of the weather, including temperature and precipitation. In general, low pressure brings clouds and precipitation, and vice versa. Although the best model uses maximum pressure as its input variable, it should be noted that the performances of other models are not distinctly worse (Table 4). For instance, the model with mean air temperature (T_mean_) as an input variable shows not only fitted RMSE that is only 2% higher than the one with maximum pressure, but also a better AIC value. In this model, the influenza incidences are positively associated with the mean air temperature. Whereas the model with minimum or maximum relative humidity shows that influenza is positively associated with humidity (both minimum and maximum). These results are in agreement with the findings that dry and cold condition enhances influenza transmission.

Both Hong Kong and Maricopa County have comparable temperatures, where it is generally warm throughout the year. Nevertheless, Maricopa County is characterized more by desert climate, where it is drier and hotter (especially in the summer) than Hong Kong's subtropical climate where rainfall is much more frequent. Therefore, the driving factors for influenza transmission may not be the same as it is reflected in our analysis. Our results show that Hong Kong influenza cases are associated with rainfall (Table 3), whereas it is not the case in Maricopa County. We had included rainfall in the model for Maricopa County but the results were unsatisfactory (not shown). The association of rainfall with influenza only in Hong Kong corroborates the finding that contact transmission is more predominant in the tropics [18], with a reasoning that rainfall induces social behavior that promotes contact transmission. Other environmental factors influencing influenza transmission seems to be common for both regions–temperature and relative humidity, although measured in different indicators for both regions–which enhance virus survivorship and aerosol transmission.

In this paper we have demonstrated the use of environmental/meteorological variables–as obtained from the satellite and ground stations–and the influenza biosurveillance data through a mathematical model, to assess the factors associated with influenza incidences. We have shown the prediction capability of the models, as measured by the RMSE of prediction dataset (Table 1 and Table 3), to forecasts the next influenza season. Presently we use the one-step ahead forecast in calculating the future influenza cases. In reality this may only be possible for cities with more advanced computer-based surveillance systems such as New York City and Hong Kong. Most of the models developed here depend on the past one to two weeks influenza cases. A more realistic approach is to predict the influenza cases using more than one-step ahead forecasts. This means that future forecasts are calculated using previously predicted number of cases instead of using the actual cases from the surveillance data (as in one-step ahead approach). However, one caveat to this approach is that more data is needed, since model selection will be based not only on the RMSE of the fitting dataset but also on the prediction dataset. When the model incorporates seasonality, the number of data that can be used for fitting process decreases significantly, especially in this study where the seasonal period is 52 weeks. In this approach one would divide the data into three: (i) for fitting process, where the coefficients are estimated, (ii) forecasting process, where future values are calculated using the predicted values, and the goodness of fit statistics will typically be used in the model selection and (iii) for validation process, to ensure that the model does not behave erratically. Thus the models in this study are a first step towards developing an early warning system for influenza.

## Materials and Methods

### Materials/Data

This study uses weekly count of laboratory-confirmed influenza viruses in two regions, Hong Kong and Maricopa County. We obtained Hong Kong influenza count between January 2005 to September 2008, from weekly influenza report as published by the Department of Health, Government of the Hong Kong Special Administrative [20]. Maricopa County influenza data, spanning October 2004–March 2009, were obtained from the weekly report by the Maricopa Department of Public Health [21]. Maricopa County flu reports are available during the flu season that usually begins from week 40 and ends at week 17 of the following year. We consequently assumed that flu counts outside the season to be zero.

Climatic and meteorological parameters were collected from two primary sources: ground-based and satellite-derived measurements. From the Hong Kong Observatory [22] we retrieved daily meteorological observations including temperature (maximum, mean, minimum), mean dew point temperature, mean relative humidity, global solar radiation and total evaporation. Maricopa County daily climatic observations were acquired from The Flood Control District of Maricopa County [23], where we aggregated data from 32 stations. This data includes daily mean air temperature, dew point (minimum, mean and max), relative humidity (minimum and maximum), maximum wind speed, air pressure (minimum and maximum) as well as maximum solar radiation.

Furthermore, we obtained remotely-sensed daily rainfall measurements for both Hong Kong and Maricopa County, from the instruments embarked on the Tropical Rainfall Measuring Mission (TRMM) [24], [25]. TRMM is a collaborative mission between NASA and Japan Aerospace Exploration Agency. The goal of the mission is to monitor and study tropical rainfall in order to improve the understanding of the water cycle in the climate system. Of the five instruments onboard the spacecraft, the Precipitation Radar and the TRMM Microwave Imager have the most direct relationship to measuring precipitation. As a follow-on mission for TRMM, the Global Precipitation Mission [26], [27], which is a multinational mission involving a constellation of satellites, will be launched in 2013. The TRMM data was retrieved using the GES-DISC Interactive Online Visualization ANd aNalysis Infractructure (Giovanni) as part of the NASA's Goddard Earth Sciences (GES) Data and Information and Services Center (DISC) [28]. In addition to TRMM data as rainfall measurements, we extracted Daily Land Surface Temperature (LST) from the MODerate resolution Imaging Spectroradiometer (MODIS) data set [29]. Both Terra and Aqua missions of NASA's Earth Observing System carry this instrument. The instrument has 36 bands spanning from the visible to the long wave infrared spectra, from which information related to disease transmission can be extracted. The importance of temperature and precipitation–as environmental determinants in the infectious disease transmission–have rendered TRMM and MODIS significant roles in remote sensing-based disease surveillance which has been demonstrated elsewhere [30], [31].

## Analysis

The weekly influenza count data was divided into two: one set was used in the fitting process (parameter estimation), and another for prediction. We took the observations in the latest 1 year as the prediction period. Out of 194 observations in Hong Kong influenza data, we used 155 points for fitting and 39 for prediction. For Maricopa data, the fitting set consists of 159 observations and the prediction set has 75.

The influenza time series that we analyzed in this study is characterized by a strong autocorrelation, a property that commonly violates the ordinary linear regression. Thus in order to account for the autocorrelation behavior, we employed a class of time series technique namely Auto Regressive Integrated Moving Average (ARIMA) [32], [33]. We first developed a univariate ARIMA model, where the response series depends only on its past values and some random shocks, followed with multivariate ARIMA with the environmental parameters as input series/covariates. In the following we will briefly delineate the Box-Jenkins approach for ARIMA modeling that is used throughout this study.

ARIMA is based on the assumption that the response series is stationary, that is the mean and variances of the series are independent of time. Stationarity can be achieved by differencing the series, or transforming the variable so as to stabilize the variance or mean. In our analysis we took the logarithmic transformation to reduce the variances of the influenza time series, and subsequently differenced the series until it is stationary. Once the response series is stationary, we examined the Autocorrelation function (ACF) and Partial Autocorrelation Function (PACF) to determine the initial autoregressive (AR) and moving average (MA) order. An ARIMA model is notated as ARIMA(*p,d,q*), where *p* indicates the AR order, *d* the differencing order and *q* the MA order. An ARIMA model that incorporates seasonality is referred as SARIMA(*p,d,q*)(*P,D,Q*) where *P,D* and *Q* indicate the seasonal order of AR, differencing, and MA, respectively. Since the influenza time series is recorded as weekly observations, the seasonality period is 52.

Based on the ACF and PACF we fitted several ARIMA models with varying AR and MA orders. In the fitting process, the AR and MA coefficients were estimated using conditional least square method. The residuals were further inspected for autocorrelation through ACF and PACF. Models with autocorrelated residuals were discarded, else goodness of fit were examined through calculated Akaike's Information Criterion (AIC) and the Mean Square Error (RMSE). The resulting model was subsequently used to forecast (1 step ahead) the latest influenza season, and the associated RMSE were calculated.

Once we developed and selected a univariate ARIMA, we investigated the effect of the environmental variables and the corresponding lags on the influenza cases. Note that the daily environmental data were converted into weekly resolution by taking the average. The environmental series were first pre-whitened. In other words, we applied univariate ARIMA modeling, as previously discussed, such that the environmental series no longer characterized by autocorrelation. Subsequently, cross-correlations function (CCF) between the pre-whitened environmental series and the influenza cases was then calculated so as to identify the lags to be included in the model. The significance of the cross-correlations was assessed on the basis of its two standard error limits (significant at 0.05 level). Environmental variables that did not exhibit significant cross-correlations with the influenza cases were excluded from further analysis.

Similar to univariate ARIMA fitting process, we further estimated the coefficients of the AR and MA terms as well as the lagged environmental variable. In this study, (S)ARIMA model that incorporates environmental input series is referred as (S)ARIMAX. The environmental input series were first included one at a time before combining them together. Estimated coefficients with p-values greater than 0.05 are excluded when possible and the model was re-fitted.

All ARIMA modeling and the corresponding statistical tests were performed using SAS software, Version 9.1.2 of the SAS System for Windows (SAS Institute, Inc., Cary, NC).
